# Curcumol Undermines SDF-1*α*/CXCR4/NF-*κ*B Signaling Pathway to Suppress the Progression of Chronic Atrophic Gastritis (CAG) and Gastric Cancer

**DOI:** 10.1155/2022/3219001

**Published:** 2022-09-16

**Authors:** Xuehui Ma, Lingjing Kong, Wen Zhu, Yongli Wang, Zhengbo Zhang, Yaozhou Tian

**Affiliations:** ^1^Department of Preclinical, Wuxi Hospital of Traditional Chinese Medicine, Wuxi Hospital Affiliated to Nanjing University of Chinese Medicine, Wuxi, China; ^2^Department of Gastroenterology, Wuxi Hospital of Triditional Chinese Medcine, Wuxi, China; ^3^Department of Gastroenterology, Jiangsu Province Hospital on Integration of Chinese and Western Medicine, Nanjing, China

## Abstract

CAG is the most common precancerous disease of gastric cancer, which belongs to a kind of chronic gastritis. CAG is in close association with gastric cancer, which makes itself a critical node clinically in cancer prevention and treatment. Curcumol is a main active monomer in Fuzheng Huowei decoction, which has the properties of antioxidant, antiviral, and antitumor. In this study, the expression of SDF-1*α*/CXCR4/NF-*κ*B was detected by in vivo and in vitro methods. Then, we found that the expressions of NF-*κ*B, SDF-1*α*, CXCR4, and p-NF-*κ*B were decreased in the curcumol treatment group. Curcumol inhibited gastric cancer cells' viability, migration, and invasion and induced their apoptosis. After adding the lentivirus overexpressing SDF-1*α* to the curcumol treatment group, it was found that SDF-1*α*, CXCR4, NF-*κ*B, and p-NF-*κ*B protein expressions were all increased, and the effect of curcumol on gastric cancer cells was reversed. In the nude mouse experiment, the tumor volume in the curcumol + SDF-1*α* group was the largest, and the tumor volume in the Fuzheng Huowei decoction + NC group was the smallest. In conclusion, curcumol effectively protects gastric tissue and inhibits the viability of gastric cancer cells, and curcumol regulates SDF-1*α*/CXCR4/NF-*κ*B to play a therapeutic role in chronic atrophic gastritis and gastric cancer.

## 1. Introduction

Major clinical manifestations of CAG are abdominal pain, abdominal distention, anorexia, and fatigue. It is a common digestive system disease in clinical practice. Although modern medical treatment of CAG has progressed rapidly, there is still no specific treatment in clinical practice. Clinical practice has confirmed the efficacy of traditional Chinese medicine (TCM) in the treatment of CAG precancerous lesions [1, 2]. TCM can not only improve clinical symptoms but also control and block the progression of CAG and improve gastric mucosal precancerous lesions, thereby reducing the incidence and mortality of gastric cancer [3, 4].

The stimulation of inflammatory factors promotes the infiltration of inflammatory cells, activates cytokines, changes the cellular microenvironment, and maintains chronic inflammation in the stomach [5, 6]. Changes in the microenvironment caused by chronic stimulation can further induce DNA damage, increase the frequency of gene mutations, and induce cell carcinogenesis, leading to gastric cancer. Curcumol is capable of reducing certain serum inflammatory factors level (IL-2, hs-CRP, TNF-*α*), and plays an antiatherosclerotic effect [7, 8]. Studies have verified curcumol's inhibitory effect on the inflammatory cytokines in the RAW246.7 mouse macrophages stimulated by cigarette extract by downregulating in vitro NF-*κ*B signaling pathway, and its suppressive effect on the inflammatory cytokines in hepatic stellate cells has been also confirmed [9]. Curcumol also has the biological function of inhibiting macrophage inflammation induced by lipopolysaccharide in RAW246.7 mice via the AP-1 pathway [9].

On the basis of previous research, this study was committed to exploring curcumol's therapeutic effect on CAG and gastric cancer, to explore its possible mechanism of action from the perspective of anti-inflammatory, taking the SDF-1/CXCR4 axis as an entry point to clarify the target of its role. Through the research, we will provide research data for curcumol in the treatment of CAG and gastric cancer.

## 2. Materials and Methods

### 2.1. Preparation of Chronic Atrophic Gastritis Animal Model

SD rats were adaptively reared for one week and then grouped, with 6 rats in each group. After grouping, a rat model of chronic atrophic gastritis was established: 20 mmol/L sodium deoxycholate solution was freely drunk, and 60% ethanol (2 ml/rat) was intragastrically administered once every 10 days. The gastric tissue was taken for detection 12 weeks later. After modeling, the rats were split up into three groups with 6 rats in each group. The treatment group was given intragastric administration of 20 mg/kg curcumol every day, and the model group was given the same amount of normal saline for 28 consecutive days. After modeling, those rats were grouped with 6 rats in each group. Animal experiments were authorized by the Animal Ethics Committee of Wuxi Hospital of Traditional Chinese Medicine (SSBDW2021042601).

### 2.2. Tumor Formation Test in Nude Mice

30 nude mice were injected with SGC7901 cells to establish a transplanted tumor model of gastric cancer. The tumor size and body weight were measured twice a week. After the tumors grew to about 0.5 cm in length, they were divided into 5 groups randomly, with 6 animals in each group, namely, the model control group, the curcumitol treatment group, and the curcumol treatment group + SDF-1*α* overexpression AAV group, Fuzheng Huowei decoction treatment group, and Fuzheng Huowei decoction + SDF-1*α* overexpression AAV group. Curcumol and Fuzheng Huowei decoction were administered by gavage, and AAV was intratumoral injection. The diet, defecation, body weight, death, and mental status of the mice were recorded every day; the tumor volume was reckoned twice a week.

### 2.3. HE Staining

Samples were washed with PBS and fixed with 4% paraformaldehyde. After dehydration, the tissue was cut into slices with a thickness of 2.5 *μ*m. After baking and dewaxing, the tissue was stained with hematoxylin for 5 minutes. Samples were destained with 1% hydrochloric acid alcohol to remove excess hematoxylin staining solution in the cytoplasm. Samples were stained in eosin staining solution for 2 min and then were washed with tap water for about 1 min.

### 2.4. Immunochemistry

Samples were dewaxed with xylene and graded ethanol. Antigen retrieval was then performed by soaking in 0.01 mol/L citrate buffer (pH 6.0). Sections were then immersed in 3% hydrogen peroxide to block endogenous catalase. Block with 5% goat serum in a humidified chamber at room temperature. The primary antibody was added and incubated overnight at 4°C. After washing 3 times, sections were treated with the secondary antibody and incubated at 37°C for 1 h. Sections were then washed and counterstained with hematoxylin, then dehydrated and mounted. Antibodies used in this study were listed as follows: NF-*κ*B (1 : 100, 10745-1-AP, Proteintech, China), SDF-1*α* (1 : 50, ab155090, Abcam, England), CXCR4 (1 : 100, Ab19035, Abclonal, China).

### 2.5. MTT Assay for Cell Viability

SGC7901 cells were fetched from the Chinese Academy of Sciences. A complete medium containing 10% FBS and 1% penicillin-streptomycin was prepared. The cells were seeded in 96-well plates. 20 *μ*L of 5 mg/mL MTT solution was added to each well. Incubate the culture plate in the incubator for 4 h. Then, 150 *μ*L of dimethyl sulfoxide was added to each well, which was shaken at low speed on a shaker for 10 min for the full dissolution of the crystals. The absorbance of each well was reckoned at OD490 nm of an enzyme-linked immunosorbent assay.

### 2.6. Detection of Apoptosis by Flow Cytometry

SGC7901 cells were divided into the following groups: control group, curcumol treatment group 100 mg/L (48 h), and Fuzheng Huowei decoction treatment group 100 mg/L (48 h). The apoptosis of SGC7901 cells was assessed by flow cytometry. Cells were centrifuged at 300 g for 5 min, and the supernatant was discarded. The cells were collected, which were washed once with PBS and gently resuspended for counting. Cells were washed once with PBS with the supernatant discarded after centrifugation. The mixture was added with 100 *μ*L of Annexin V binding buffer working solution and then was added with 2.5 *μ*L of Annexin V-APC and 2.5 *μ*L of DAPI staining solution. Cells were incubated at room temperature for 15 min avoiding light.

### 2.7. Transwell Assay

Cell migration assay: 1 mL of EDTA-containing trypsin was added to digest SGC7901 cells. The cell density was adjusted to 5 × 10^5^/mL. 100 *μ*L of cell suspension was added to each well and stimulated by drug addition, 500 *μ*L of complete medium containing 20% FBS was added to the lower chamber and incubated in a 37°C incubator for 24 h. The transwell chamber was taken out and the culture medium in the well was discarded, washed twice with calcium-free PBS, fixed with 4% paraformaldehyde for 20 min, and stained with 0.1% crystal violet for 20 min after washing with PBS. Then the chamber was gently wiped off with a damp cotton ball. Unmigrated cells in the upper layer were washed 3 times with PBS.

Cell invasion assay: the 24-well plate and 1 mL pipette tip were placed in the refrigerator to precool for 30 min. Afterward, 300 *μ*L serum-free medium was taken, then 60 *μ*L matrigel was added, mixed well, 100 *μ*L of which was added to the upper chamber and incubated in a 37°C incubator for 5 h. 1 mL of EDTA-containing trypsinized SGC7901 cells was added and the cell density was adjusted to 5 × 10^5^/mL. The matrigel was washed once with serum-free medium, 100 *μ*L of cell suspension was added to each well, and 500 *μ*L of complete medium containing 20% FBS was added to the lower chamber, and incubated in a 37°C incubator for 24 h. The transwell chamber was taken out with the culture medium in the well discarded, which was washed twice with calcium-free PBS, fixed with 4% paraformaldehyde for 20 min, and stained with 0.1% crystal violet for 20 min after washing with PBS. The chamber was gently wiped off with a damp cotton ball Unmigrated cells in the upper layer were washed 3 times with PBS.

### 2.8. Western Blot

RIPA lysis buffer was added to the protein expression samples by western blot, and the protein concentration was measured by BCA kit to prepare protein loading buffer. After 80 V runs through the concentrated gel, convert the voltage to 120 V, and wait until the bromophenol blue runs to the bottom of the rubber plate and just does not run out. The current was adjusted to a constant current of 250 mA and transferred for 1 h. Then, the PVDF membrane was washed in TBST for 1 min and then blocked with 5% nonfat milk blocking solution for 1 h at room temperature. The primary antibody was diluted in 1 : 1000 with primary antibody diluent, which was incubated with the PVDF membrane at 4 degrees overnight. The secondary antibody was diluted to a certain concentration (1 : 2000) with a blocking solution and then incubated at room temperature for 1 h. Mix the ECL exposure solution at 1 : 1 with solution A: solution B and cover the entire film evenly, react for 1 min, and put it into the exposure meter for exposure detection. Antibodies used in this study were listed as follows: NF-*κ*B (1 : 500, 10745-1-AP, Proteintech, China), SDF-1*α* (1 : 500, ab155090, Abcam, England), CXCR4 (1 : 200, Ab19035, Abclonal, China), p-NF-*κ*B (1 : 500, 3033T, CST, USA).

## 3. Results

### 3.1. Curcumol Suppressed the NF-*κ*B, SDF-1*α*, and CXCR4 Expressions in the CAG Model

**Figure 1:**
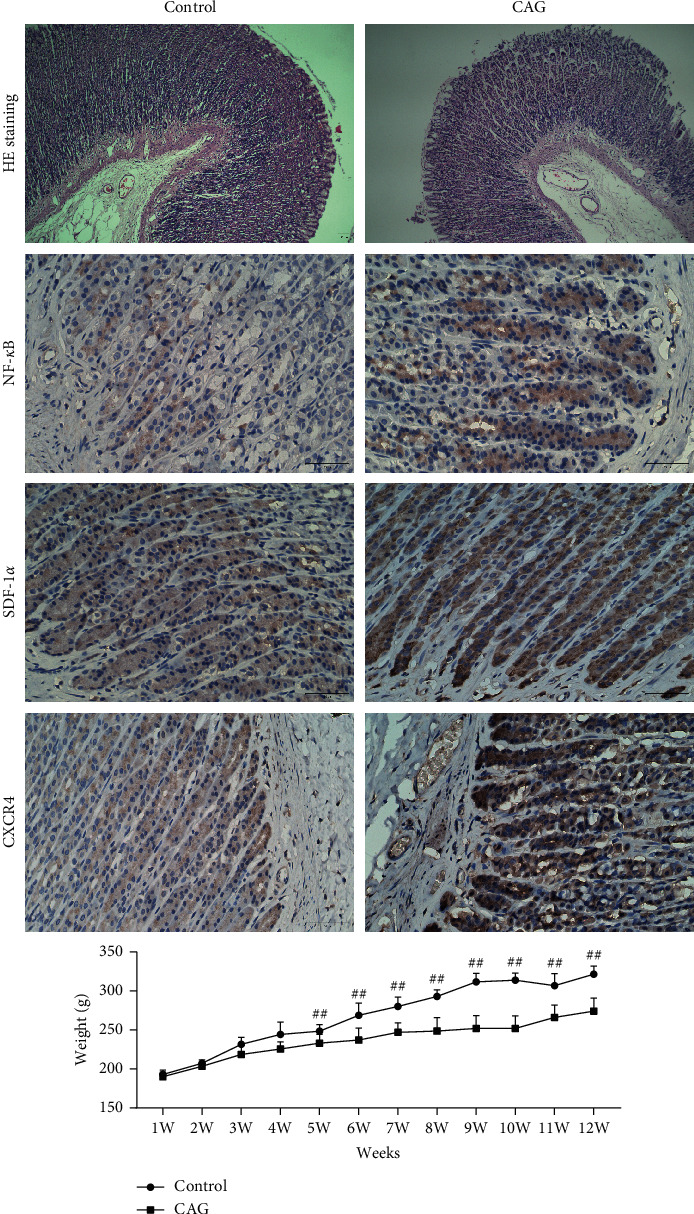
Construction and detection of the CAG model. HE staining and immunohistochemistry were used to detect the tissue structure and marker protein expression of CAG model animals. Body weight detection of two groups of animals in 1–12 weeks.

**Figure 2:**
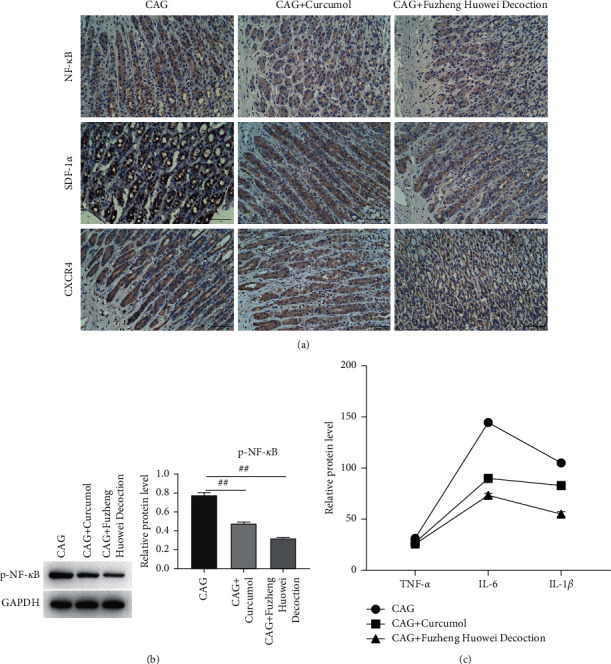
Curcumitol regulates protein expression in the CAG model. (a) Immunohistochemical detection of NF-*κ*B, SDF-1*α*, and CXCR4 expression. (b) Western blot detection of p-NF-*κ*B expression. (c) ELISA detection of TNF-*α*, IL-6 and IL-1*β* expression.

In this study, we first used ethanol to prepare the CAG animal model, and then detected the tissue structure and markers of the model animal by HE staining and immunohistochemistry. The results unveiled that the gastric tissue in the CAG model group was disordered in morphology, irregular in tissue structure, and infiltrated by inflammatory cells. In comparison with the control group, the NF-*κ*B, SDF-1*α*, and CXCR4 expressions in the CAG model group were significantly increased (Figure 1). The body weights of the two groups of rats were measured for 1–12 weeks, and it was found that from the fifth week, the body weight of the rats in the CAG model group was lower than that in the control group. These results unveiled that CAG has obvious harm to the gastric tissue and body weight of animals. Then, after adding curcumol and the positive drug Fuzheng Huowei decoction to the model group respectively, we detected protein expression by immunohistochemistry and found that compared with the model group, NF-*κ*B, SDF-1*α*, The expression levels of CXCR4 were all decreased (Figure 2(a)). Results of western blot demonstrated that expression of p-NF-*κ*B was conspicuously reduced in the group added with curcumitol or the positive drug (Figure 2(b)). The results of ELISA showed that in contrast to the model group, the IL-6 and IL-1*β* expressions in curcumitol treatment or positive drug group manifested a decreasing trend, and the expression level of TNF-*α* was similar (Figure 2(c)).

### 3.2. Curcumol Inhibited the Viability, Migration, and Invasion of Gastric Cancer Cells

**Figure 3:**
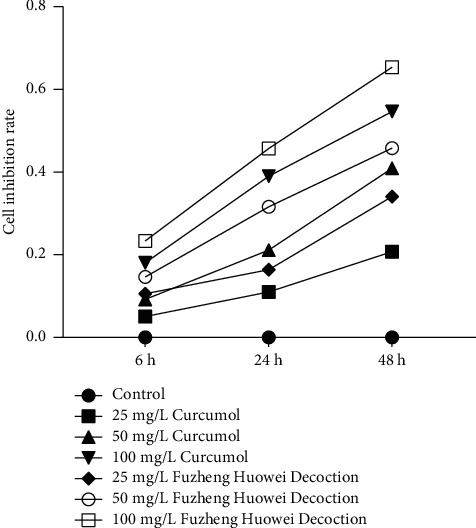
MTT assay to detect the viability of gastric cancer cells.

Next, we tested the regulatory function of curcumol in gastric cancer cells cultured in vitro. The results showed that 100 mg/L Fuzheng Huowei decoction had the highest cell inhibition rate at 48 h, followed by 100 mg/L curcumol for 48 h (Figure 3). Therefore, 100 mg/L (48 h) of the curcumol treatment group and 100 mg/L (48 h) of the Fuzheng Huowei decoction treatment group were selected for follow-up experiments.

**Figure 4:**
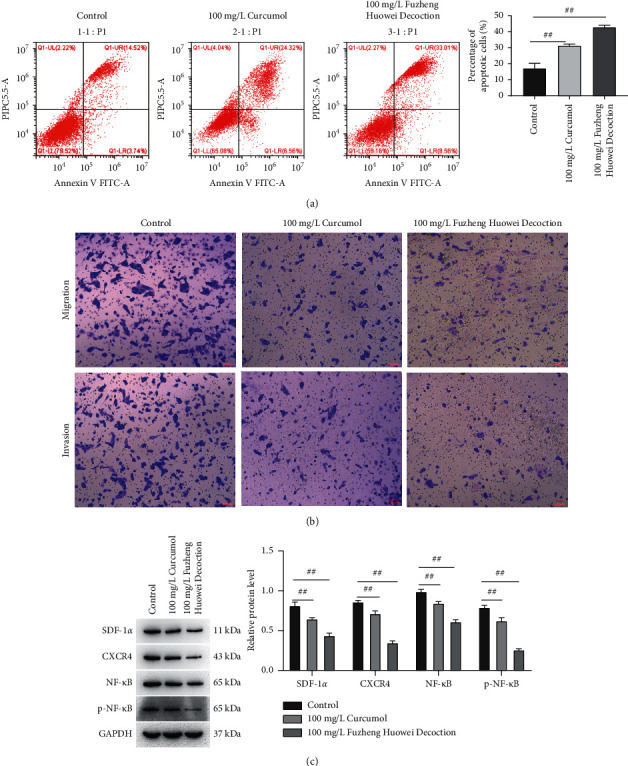
The regulation of curcumol on apoptosis, migration, and invasion of gastric cancer cells. (a) Flow cytometry assay on cellular apoptosis. (b) Transwell detection on gastric cancer cells' migration and invasion. (c) Western blot detection of SDF-1*α*, CXCR4, NF-*κ*B, and p-NF-*κ*B protein expression in gastric cancer cells.

It was found that the apoptosis rate of gastric cancer cells in the control group, curcumol treatment group, and Fuzheng Huowei decoction treatment group were 17.23%, 31.37%, and 42.95%, respectively (Figure 4(a)). A Transwell assay was conducted for the detection of gastric cancer cells' migration and invasion, and it was found that after treatment of gastric cancer cells with curcumol and Fuzheng Huowei decoction, the number of migratory and invasive cells was significantly reduced (Figure 4(b)). Further testing showed that the SDF-1*α*, CXCR4, NF-*κ*B, and p-NF-*κ*B protein expressions in gastric cancer cells were declined after a treatment with curcumol and the positive drug Fuzheng Huowei decoction (Figure 4(c)).

### 3.3. SDF-1*α* Reverses the Inhibitory Effect of Curcumol on Gastric Cancer Cells

**Figure 5:**
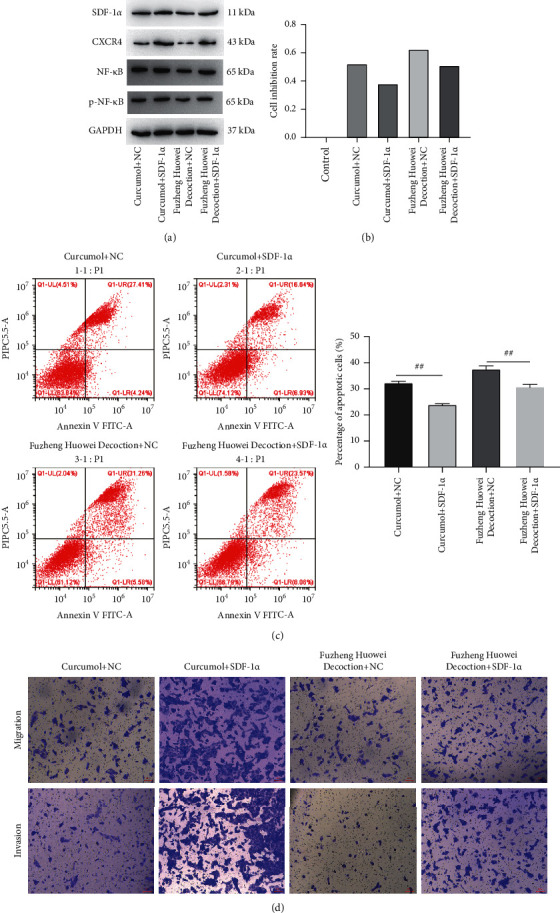
SDF-1*α* reverses the inhibitory effect of curcumol on gastric cancer cells. (a) Western blot detection onto SDF-1*α*, CXCR4, NF-*κ*B, and p-NF-*κ*B protein expression. (b) MTT detection on cellular viability. (c) Flow cytometry detection on apoptosis rate. (d) Transwell detection on gastric cancer cells' migration and invasion.

In order to prove that curcumol exerts its regulatory function by regulating the SDF-1*α*/CXCR4/NF-*κ*B axis, this study cotreated gastric cancer cells with SDF-1*α* overexpressing lentivirus and curcumol. The results showed that SDF-1*α*, CXCR4 protein expressions, and NF-*κ*B were all increased after overexpression of SDF-1*α* (Figure 5(a)). The results of the MTT assay showed that overexpression of SDF-1*α* could reverse the inhibitory effect of curcumol or the positive drug Fuzheng Huowei decoction on gastric cancer cells (Figure 5(b)). The results of flow cytometry also demonstrated that when compared with the empty virus group, the apoptosis rate of cells overexpressing SDF-1*α* was significantly reduced (Figure 5(c)). In addition, the numbers of gastric cancer cell invasion and migration were also significantly increased (Figure 5(d)).

**Figure 6:**
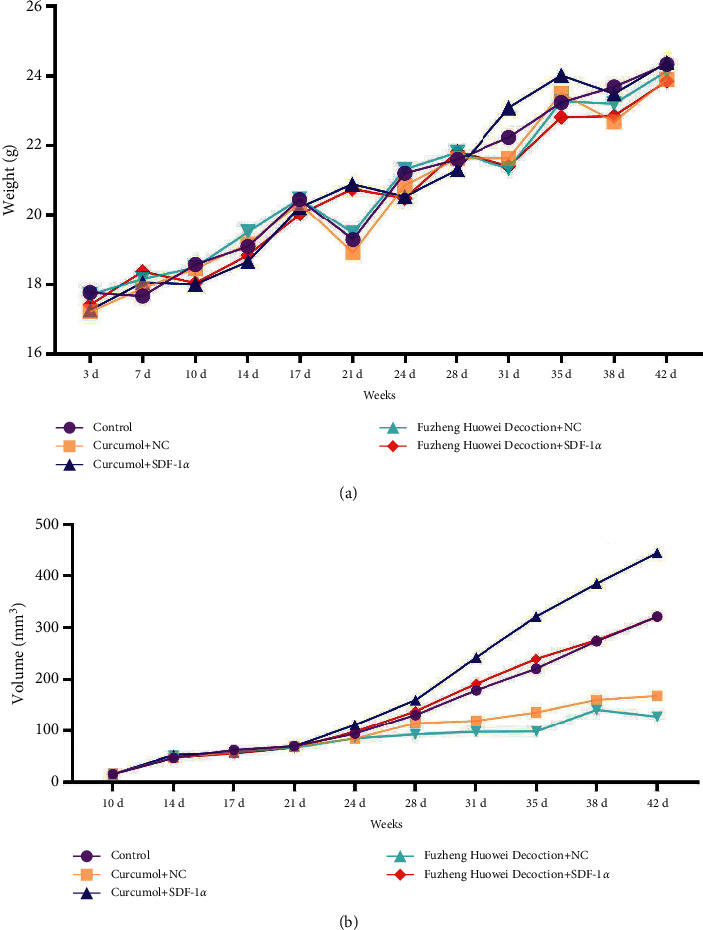
Results of body weight and tumor volume determination in nude mice tumorigenic test. (a) Results of body weight determination of nude mice. (b) Results of tumor volume detection in nude mice.

**Figure 7:**
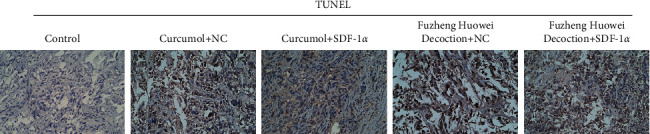
TUNEL detected apoptosis in tumors after treatment in each group.

Finally, we further verified the regulatory effect of SDF-1*α* on gastric cancer cells by nude mouse tumorigenesis assay. The results showed that the body weight of nude mice in the control group, curcumol + NC group, curcumol + SDF-1*α* group, Fuzheng Huowei decoction + NC group, and Fuzheng Huowei decoction + SDF-1*α* group did not change significantly during the test period (Figure 6(a)). However, there were significant distinctions in tumor volume among groups. The tumor volume in the curcumol + SDF-1*α* group was the largest, and the tumor volume in the Fuzheng Huowei decoction + NC group was the smallest (Figure 6(b)). TUNEL was used to detect the apoptosis of tumors in each group after treatment, and it turned out that the positive rate of TUNEL in overexpressing the SDF-1*α* group was also lower than in the corresponding empty virus group (Figure 7).

## 4. Discussion

Treatment and reversal of CAG are important strategies for controlling gastric cancer. Years of experimental studies have proved that traditional Chinese medicine is endowed with advantages in CAG treatment. Traditional Chinese medicine treatment is capable of improving the clinical symptoms of patients and effectively preventing or even reversing precancerous lesions [10]. Although the mechanism of CAG is not very clear, in recent years, the experimental research of CAG in the field of molecular biology and genetics has been deepened. Compared with synthetic compounds, traditional Chinese medicine has less toxicity and has various regulating effects on the human body. This study confirmed in the previous clinical study that Fuzheng Huowei decoction can effectively relieve the clinical symptoms of CAG patients, improve histological lesions, and promote mucosal repair. This study showed that curcumol inhibited the NF-*κ*B, SDF-1*α*, and CXCR4 expressions in the CAG model and gastric cancer cells, and also the viability, migration, and invasion of gastric cancer cells, inducing their apoptosis in animal level and in vitro cultured cells.

NF-*κ*B is an important regulatory factor regulating the initiation and amplification of inflammatory responses. Additionally, studies showed that the NF-*κ*B inflammatory signaling pathway is often in a state of persistent abnormal activation in gastric cancer [11, 12]. It has been found that *Helicobacter pylori* infection and the inflammatory response caused by environmental factors constitutively activate NF-*κ*B [13–15]. The abnormally activated NF-*κ*B signaling further promotes the expression of downstream molecules, including cytokines, cytokine receptors, and cellular adhesion factors, regulates the inflammatory response, and participates in the onset and development of gastric cancer [16]. It was found that the expression of NF-*κ*B gradually increased with the development of gastric cancer [17]. These studies indicated that the NF-*κ*B-mediated inflammatory signaling pathway is increased during the development of gastric cancer. In this study, we found that NF-*κ*B is in close association with the development of CAG and gastric cancer. The contents of NF-*κ*B and p-NF-*κ*B in patients were significantly decreased by curcumol turmerol treatment, which inhibited inflammation, CAG, and gastric cancer.

As intercellular signaling molecules, they can participate in immune response regulation, tissue injury repair and mediating inflammatory response, and other processes [18, 19]. There are dozens of chemokines that have been discovered so far, which are categorized into four families: CXC, CC, CX3C, and C. SDF-1 is a stromal cell-derived factor and belongs to the chemokine CXC subfamily. CXCR4 is a specific receptor of SDF-1*α*, which is involved in tumor growth, invasion, and metastasis [20, 21]. The SDF-1*α*/CXCR4 axis regulates inflammatory responses in diseases such as chronic skin inflammation, osteoarthritis, acute peritonitis, and colon and oral squamous cell carcinoma. It has also been reported in the literature that the SDF-1*α*/CXCR4 axis promotes the migration and invasiveness of human papillary thyroid cancer cells by the NF-*κ*B signaling pathway. In addition, in a clinical sample study of gastric cancer, it was reported that with the progression of gastric cancer, the positive expression rate of SDF-1 (17% in superficial gastritis, 50% in precancerous lesions group, and 85% in gastric cancer group) and CXCR4 (8% in superficial gastritis group, 54% in gastric cancer group and 81% in gastric cancer group) was gradually increased, which was also consistent with the expression trend of NF-*κ*B in gastric cancer progression [22]. In this paper, we confirmed that curcumol exerts anti-inflammatory activity by regulating the SDF-1/CXCR4 axis and suppressing the NF-*κ*B pathway during the development of gastric cancer. We have demonstrated that curcumol can simultaneously reduce the expression of NF-*κ*B, SDF-1*α* and CXCR4 in CAG animal models and gastric cancer in vivo and in vitro experiments.

In the last decades, Chinese medicine has made great progress in the treatment of CAG and gastric cancer, especially the application of traditional Chinese medicine and ethnic medicine injection, which has good clinical efficacy. Curcuma oil is a large variety and classic traditional Chinese medicine and ethnic medicine injection. It has been used in the clinical treatment of tumors for a long time, and the effect is excellent. The current study found that Curcuma alcohol is the primary antitumor active component of Curcuma oil. In this study, we found that curcumol could reduce the SDF-1*α*/CXCR4/NF-*κ*B protein expression in gastric cancer cells. When SDF-1*α* was overexpressed, the protein expressions of SDF-1*α*, CXCR4, and NF-*κ*B were all increased, which would offset the inhibition from curcumol on gastric cancer cells. Through the research on this subject, it is proved that curcumol can treat CAG and reverse the progression of gastric cancer by regulating the SDF-1/CXCR4 axis. This study provides research data for curcumol in the treatment of CAG and gastric cancer and provides an important basis for the improvement of the antitumor mechanism of traditional Chinese medicine.

## 5. Conclusion

Curcumol suppresses the viability, migration, and invasion of gastric cancer cells and induces apoptosis of gastric cancer cells. Curcumol treatment can reduce NF-*κ*B, p-NF-*κ*B, SDF-1*α*, and CXCR4 expression. In the nude mouse experiment, the tumor volume in the curcumol + SDF-1*α* group was the largest, and the tumor volume in the Fuzheng Huowei decoction + NC group was the smallest. Curcuma effectively protects gastric tissue and inhibits gastric cancer cell viability. Curcumol regulates SDF-1*α*/CXCR4/NF-*κ*B to play a therapeutic role in chronic atrophic gastritis and gastric cancer.

## Data Availability

The data used to support the findings of this study are included within the article.
